# An artificial intelligence model for predicting an appropriate mAs with target exposure indicator for chest digital radiography

**DOI:** 10.1038/s41598-025-96947-y

**Published:** 2025-04-08

**Authors:** Jia-Ru Lin, Tai-Yuan Chen, Yu-Syuan Liang, Jyun-Jie Li, Ming-Chung Chou

**Affiliations:** 1 Department of Radiology, Kaohsiung Armed Force General Hospital, Kaohsiung, Taiwan; 2 Department of Radiology, Chi Mei Medical Center, Tainan, Taiwan; 3 Graduate Institute of Medical Sciences, Chang Jung Christian University, Tainan, Taiwan; 4 Department of Radiation Oncology, E-DA Hospital, Kaohsiung, Taiwan; 5 Department of Medical Imaging and Radiological Sciences, Kaohsiung Medical University, Kaohsiung, Taiwan; 6 Department of Medical Research, Kaohsiung Medical University Hospital, Kaohsiung, Taiwan; 7 Biomedical Artificial Intelligence Academy, Kaohsiung Medical University, Kaohsiung, Taiwan

**Keywords:** Machine learning, mAs, Reached exposure, Chest radiography, Radiography, Biomedical engineering

## Abstract

In digital radiography, image quality is synergistically affected by anatomy-specific examinations, exposure factors, body parameters, detector types, and vendors/systems. However, estimating appropriate exposure factors before radiography with optimized image quality without overexposure or underexposure to patients is difficult. Thus, there is an unmet need to establish a model to predict appropriate mAs for optimizing image quality before radiography. This study aimed to establish a machine learning (ML) model for predicting an appropriate current–time product (mAs) using the target exposure indicator in chest digital radiography. An anthropomorphic chest phantom was used to establish a target exposure indicator which was used to define overexposure and underexposure in the human study. This study enrolled 1,000 (M/F = 915/85) subjects who underwent regular chest radiography. The chest thickness, height, weight, body mass index, mAs, and concomitant reached exposure (REX) were recorded. To construct the prediction model, the dataset was randomly separated into training (80%) and testing (20%) sets by matching their demographic characteristics. Five ML models were trained using the training set with 10-fold cross validation, and the model performance was evaluated using the testing set with correlation coefficients, root–mean–square error, and mean average error. The phantom study showed that the average REX was 355.6 which served as the target exposure indicator. In human study, the comparisons showed that the artificial neural network (ANN) model was the most suitable for predicting both REX and mAs values. The results demonstrated that, on average, the predicted mAs values were 10% lower and 8% higher than the values determined by AEC in the overexposure (REX > 355.6) and underexposure (REX < 355.6) groups, respectively. Moreover, the predicted mAs values were further reduced in all patients when lowering the target REX values. We concluded that the ML approach was feasible for building an artificial intelligence model for predicting appropriate mAs with target exposure indicator for chest digital radiography.

**Supplementary Information:**

The online version contains supplementary material available at 10.1038/s41598-025-96947-y.

## Introduction

Chest radiography is one of the most common imaging modalities in radiology and is the first-line diagnostic tool for general chest assessments. To obtain a chest radiograph with adequate image quality, adjusting the exposure parameters, kVp and mAs, is usually mandatory based on the patient’s body parameters, including body size, weight (W), height (H), W/H, body mass index (BMI), and chest thickness (T)^1–7^. Currently, digital radiographic systems are usually equipped with flat panel detectors (FPDs) and automatic exposure control (AEC). FPDs allow a wide dynamic range of radiation exposure and have favorable combination of image quality and radiation dose^8,9^. Furthermore, AEC helps to avoid overexposure while maintaining adequate image quality^10–13^. Because AEC automatically terminates X-ray tube currents after exposure to a certain amount of radiation, it substantially avoids overexposure of patients when the upper mAs limit is too high. However, AEC may likely increase the radiation dose to patients when a thick body part is placed immediately in front of the detector^14,15^. Therefore, setting a proper upper mAs limit is crucial to avoid overexposure to patients when using AEC.

In digital radiography, the exposure parameters kVp and mAs are intimately associated with radiographic image quality^3,6,16–19^. Generally, kVp and mAs are correlated with image contrast and signal-to-noise ratio, respectively^16,20^. In chest radiography, the kVp is usually kept constant because the thorax mainly comprises lung tissue, whereas the mAs is usually determined using AEC. Therefore, mAs is the main exposure factor that affects the image quality in chest radiography. To assess physical image quality, studies have suggested using exposure indicators, such as the exposure index, reached exposure (REX), S value, log of median of histogram (lgM), and dose indicator (DI)^21–26^. While exposure indicators may be defined differently by vendors and manufacturers, they generally exhibit a linear or inverse linear relationship with detector exposure.

To maintain constant image quality, a target exposure indicator should be properly determined for each anatomy-specific examination and radiography system for clinical use^22,24^. One study suggested determining the target exposure indicator value by calculating the average exposure indicator values from repeated phantom radiography images using AEC^24^. However, radiographic image quality is synergistically affected by anatomy-specific examinations, exposure factors (i.e., kVp and mAs), body parameters (i.e., W, H, W/H, BMI, T), detector types, and vendors/systems^1–7^; therefore, estimating appropriate exposure factors before radiography without overexposure or underexposure to patients is difficult. In clinical practice, although AEC can automatically determine the mAs during radiography, positioning variations may lead to mAs variations and cause overexposure in patients^6,14,15^. Thus, there is an unmet need to establish a model to predict appropriate mAs that generates radiographs with consistent image quality before radiography.

Recently, artificial intelligence (AI) has been widely used to solve classification and prediction problems in medical applications; however, no previous study has used AI models to predict the mAs values under specific body parameters before radiography. To construct an accurate prediction model, it is crucial to obtain precise body parameters for AI models. Among these parameters, chest thickness has traditionally been measured using a caliper or ruler^2^, which is prone to errors. Inaccurate measurements of chest thickness can introduce additional bias in mAs prediction. Therefore, a previous study proposed a two-dimensional thickness measurement technique using a non-contact infrared sensor that generates a distance map based on the reflection of infrared rays between the object and the sensor^6^. The results demonstrated that the non-contact infrared sensor can accurately measure distance and had an excellent linear relationship between the actual and measured distances^6^. Therefore, measuring chest thickness with a non-contact infrared sensor may significantly enhance the accuracy and reliability of AI models in predicting appropriate mAs for chest radiography.

With the objective of developing an AI model for predicting mAs in chest radiography, this study utilized ML techniques with specific body parameters and an exposure indicator. To ensure accuracy and reliability, chest thickness was measured using a non-contact infrared sensor and combined with other body parameters such as W, H, W/H, and BMI to construct the prediction model. Additionally, the AI model incorporated the target exposure indicator derived from chest phantom radiography to predict the appropriate mAs. This comprehensive approach aimed to improve the precision and effectiveness of mAs prediction in chest radiography.

## Materials and methods

### Phantom study

**Fig. 1:**
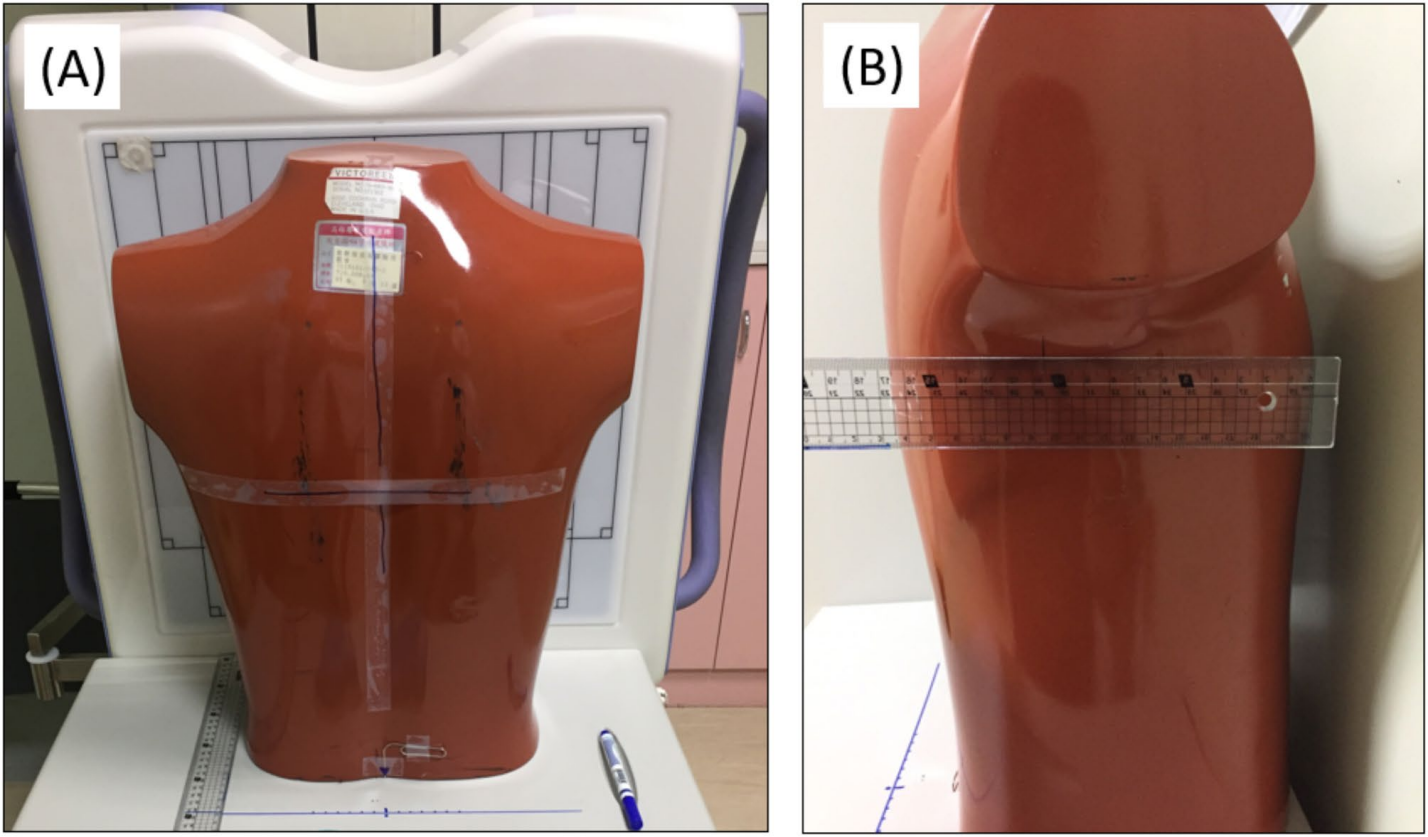
The posterior (**A**) and lateral (**B**) views of the anthropomorphic chest phantom which has a thickness of 16.5 cm and roughly represents an average male subject with height of 175 cm and weight of 74 kg.

To establish a target exposure indicator for chest radiography, an anthropomorphic chest phantom (Victoreen model 76-683-3000, Cleveland, Ohio, USA), as shown in Fig. 1, was placed at the center field of view and repeatedly exposed to acquire five radiographs using a digital radiography system (CXDI-401 C CANON; FPD-TFT; KXO-32 S TOSHIBA). The exposure parameters were 105 kVp and 200 mA, and AEC was used to automatically determine the mAs. Because the vendor-specific exposure indicator provided by the manufacturer is REX value^22^, the resulting REX values were averaged to serve as the target exposure indicator for chest radiography.

## Human study

This study was approved by the local Institutional Review Board of Kaohsiung Armed Forces General Hospital (protocol code: 106 − 028 and date of approval: June 11, 2017), and informed consent was obtained from each participant in accordance with the Helsinki Declaration. This study enrolled 1,000 consecutive (M/F = 915/85) subjects who underwent regular posterior-to-anterior chest radiography at outpatient imaging center, and informed consent was obtained from each participant. The inclusion criteria were patients aged 20 years or older, without metal implants or scoliosis, and able to undergo a standing chest radiography. The average chest thickness in the central area of about 15 × 15 cm^2^ was measured using a non-contact infrared sensor mounted on top of X-ray tube^6^, and H, W, and BMI were also recorded for the analysis. The mAs and concomitant REX values were obtained during chest radiography by using a digital radiography system (CXDI-401 C CANON; FPD-TFT; KXO-32 S TOSHIBA). Other exposure parameters were 105 kVp and 250 mA, and the source-to-image distance was maintained at 180 cm. All images were reviewed by experienced radiologists and exhibited adequate diagnostic image quality. Demographic characteristics of the enrolled patients are presented in Table 1.

**Table 1:** The demographic characteristics, exposure parameter and indicator of all enrolled subjects.

Number of patients	Age (years)	Height (cm)	Weight (kg)	W/H (kg/cm)	BMI (kg/m^2^)	Body thickness (cm)	mAs	Reached exposure (REX)
(M/F = 915/85)	25.8 ± 7.0	171.7 ± 6.6	71.4 ± 13.5	0.42 ± 0.07	24.2 ± 4.0	18.9 ± 3.7	2.74 ± 1.20	362.2 ± 59.4

The data are expressed as mean ± standard deviation.

## Machine learning

To establish a prediction model, the dataset was randomly separated into training (80%) and testing (20%) sets by matching their demographic characteristics, exposure parameter and indicator (i.e. age, gender, W, H, T, W/H, BMI, mAs, and REX) using semi-random selection^27^, as shown in Table 2. Five ML models, including an artificial neural network (ANN), support vector machine (SVM), ensemble learning with boostrap aggregation (BAG), least absolute shrinkage and selection operator (LASSO), and random forest (RF), were trained using the training set with input parameters consisting of age, sex, T, W, H, W/H, BMI, and REX. A 10-fold cross validation was performed to determine the best prediction model. Finally, the model performance was evaluated for the testing dataset using the correlation coefficients, R-square, root–mean–square error (RMSE), mean average error (MAE), and computation time between the predicted and actual mAs values, and a feature importance permutation test was performed 1000 times to evaluate the importance of features in the final model. Because the ANN model comprises various subtypes (such as FeedForwardNet^28^, FitNet^29^, CasacadeForwardNet^30,31^, and ElmanNet^32^) and SVM model employs different kernels (Linear, Gaussian, Polynomial kernels)^33^, the present study specifically selected the most optimal ANN and SVM models for comparison with other models. Moreover, the structure of ANN models was built with two hidden layers of a size of 10 neurons between input and output layers. All machine learning models were built using the Statistics and Machine Learning Toolbox of MATLAB software (MathWorks, Massachusetts, USA; https://www.mathworks.com/products/statistics.html) running on a desktop personal computer (Windows 10, Intel Core i7-3770 K CPU @ 3.50 GHz, and 16 GB RAM).

**Table 2:** The demographic characteristics, exposure parameter and indicator of training and testing datasets.

Number of patients	Age (years)	Height (cm)	Weight (kg)	W/H (kg/cm)	BMI (kg/m^2^)	Body thickness (cm)	mAs	Reached exposure (REX)
Training set (M/F = 732/68)	25.9 ± 7.3	171.7 ± 6.6	71.4 ± 13.6	0.41 ± 0.07	24.2 ± 4.1	18.9 ± 3.6	2.73 ± 1.20	361.8 ± 59.2
Testing set (M/F = 183/17)	25.5 ± 6.0	171.8 ± 7.0	71.7 ± 13.0	0.42 ± 0.07	24.3 ± 4.0	18.8 ± 4.0	2.81 ± 1.24	364.4 ± 60.5

The data are expressed as mean ± standard deviation.

After determining the final mAs prediction model, the model was further used to predict appropriate mAs by employing the target exposure indicator derived from phantom chest radiography for patients with overexposure and underexposure. The overexposure and underexposure were defined as the exposure indicator higher and lower the target exposure indicator, respectively. Finally, the predicted mAs were compared with the values determined by AEC to assess the feasibility of the model predicting appropriate mAs for those with overexposure and underexposure, respectively.

Moreover, a previous study suggested that the REX values falling within 330 ± 30 were acceptable for clinical purpose^24^, so the present study further utilized the prediction model to understand how much radiation dose could be reduced and increased for those over-exposed and under-exposed patients, respectively, under different target REX values ranging from 305.6 to 395.6. The percentage change in radiation dose between the predicted and actual mAs was defined as |predicted mAs – actual mAs|/actual mAs × 100%.

### Statistical analysis

A two-sample *t* test was performed to compare the data between two datasets and between two ML models, and a paired *t* test was performed to compare the difference between the predicted and actual mAs values derived by the prediction model and AEC. The difference was considered significant if *P* < 0.05.

## Results

The results of the phantom study showed that the mean REX value was 355.6 ± 8.0 (mean ± standard deviation) in five repeated chest phantom radiographic scans which was used as the target exposure indicator in this study. In the human study, five ML models (i.e., ANN, SVM, LASSO, BAG, and RF) were constructed to predict the mAs, and the performances of the models are listed in Tables 3, 4 and 5. The results showed that the ANN, BAG, and RF models were suitable for predicting the mAs with mean correlation coefficients > 0.9 between the predicted and actual mAs values in the testing set. Among them, the ElmanNet ANN model performed the best, with mean correlation coefficients of 0.915 ± 0.003 and 0.906 ± 0.003, R-squares of 0.838 ± 0.006 and 0.820 ± 0.003, RMSE values of 0.177 ± 0.003 and 0.187 ± 0.002, MAE values of 0.336 ± 0.004 and 0.352 ± 0.005 for the training and testing datasets, respectively. Besides, the ANN model exhibited significantly higher correlation coefficient and R-square, but lower RMSE and MAE values than those of BAG and RF models in the testing set. Although the computation time of the ANN model was longer than that of BAG and RF models, it was less than 2 s during model training and testing.

**Table 3:** The comparisons of five ML models in predicting the mAs values.

	Datasets	ElmanNet ANN	Linear SVM	BAG	LASSO	RF
Correlation coefficient	Training	0.915 ± 0.003	0.905 ± 0.000	0.956 ± 0.001	0.909 ± 0.000	0.957 ± 0.001
	Testing	0.906 ± 0.003	0.898 ± 0.000	0.901 ± 0.002	0.898 ± 0.000	0.901 ± 0.002
R-square	Training	0.838 ± 0.006	0.811 ± 0.001	0.911 ± 0.001	0.827 ± 0.000	0.912 ± 0.001
	Testing	0.820 ± 0.003	0.792 ± 0.001	0.811 ± 0.003	0.806 ± 0.000	0.811 ± 0.003
RMSE	Training	0.177 ± 0.003	0.190 ± 0.000	0.131 ± 0.001	0.183 ± 0.000	0.123 ± 0.001
	Testing	0.187 ± 0.002	0.201 ± 0.001	0.192 ± 0.001	0.194 ± 0.000	0.191 ± 0.001
MAE	Training	0.336 ± 0.004	0.352 ± 0.000	0.232 ± 0.001	0.357 ± 0.000	0.231 ± 0.001
	Testing	0.352 ± 0.005	0.372 ± 0.000	0.356 ± 0.003	0.381 ± 0.001	0.356 ± 0.003
Computation time (ms)	Training	1720.7 ± 72.6	60.0 ± 5.8	513.2 ± 44.0	1760.4 ± 39.4	457.7 ± 28.7
	Testing	10.0 ± 1.2	0.9 ± 0.1	33.9 ± 7.1	0.8 ± 0.2	29.4 ± 4.0

The data are expressed as mean ± standard deviation.

**Table 4:** The comparisons of different ANN subtypes in predicting the mAs values.

	Datasets	FeedForwardNet	FitNet	CascadeForwardNet	ElmanNet
Correlation coefficient	Training	0.911 ± 0.017	0.914 ± 0.012	0.917 ± 0.012	0.915 ± 0.003
	Testing	0.898 ± 0.010	0.898 ± 0.011	0.896 ± 0.007	0.906 ± 0.002
R-square	Training	0.825 ± 0.037	0.831 ± 0.028	0.838 ± 0.025	0.838 ± 0.006
	Testing	0.799 ± 0.020	0.800 ± 0.026	0.798 ± 0.017	0.820 ± 0.003
RMSE	Training	0.183 ± 0.018	0.196 ± 0.013	0.176 ± 0.013	0.177 ± 0.003
	Testing	0.196 ± 0.009	0.219 ± 0.064	0.200 ± 0.010	0.187 ± 0.002
MAE	Training	0.325 ± 0.011	0.333 ± 0.035	0.319 ± 0.017	0.336 ± 0.004
	Testing	0.374 ± 0.016	0.380 ± 0.028	0.379 ± 0.019	0.352 ± 0.005
Computation time (ms)	Training	300.8 ± 87.6	286.0 ± 101.1	315.5 ± 64.7	1720.7 ± 72.6
	Testing	8.6 ± 0.4	8.4 ± 0.5	9.7 ± 0.8	10.0 ± 1.2

The data are expressed as mean ± standard deviation.

**Table 5:** The comparisons of different SVM models in predicting the mAs values.

	Datasets	Linear SVM	Gaussian SVM	Polynomial SVM
Correlation coefficient	Training	0.905 ± 0.000	0.915 ± 0.000	0.933 ± 0.000
	Testing	0.898 ± 0.000	0.892 ± 0.002	0.894 ± 0.001
R-square	Training	0.811 ± 0.001	0.829 ± 0.000	0.869 ± 0.000
	Testing	0.792 ± 0.001	0.789 ± 0.004	0.798 ± 0.002
RMSE	Training	0.190 ± 0.000	0.181 ± 0.000	0.158 ± 0.000
	Testing	0.201 ± 0.001	0.202 ± 0.002	0.199 ± 0.001
MAE	Training	0.352 ± 0.000	0.277 ± 0.003	0.289 ± 0.001
	Testing	0.372 ± 0.000	0.387 ± 0.003	0.372 ± 0.001
Computation time (ms)	Training	60.0 ± 5.8	48.2 ± 2.2	2221.8 ± 946.9
	Testing	0.9 ± 0.1	1.1 ± 0.2	0.8 ± 0.2

The data are expressed as mean ± standard deviation.

**Fig. 2:**
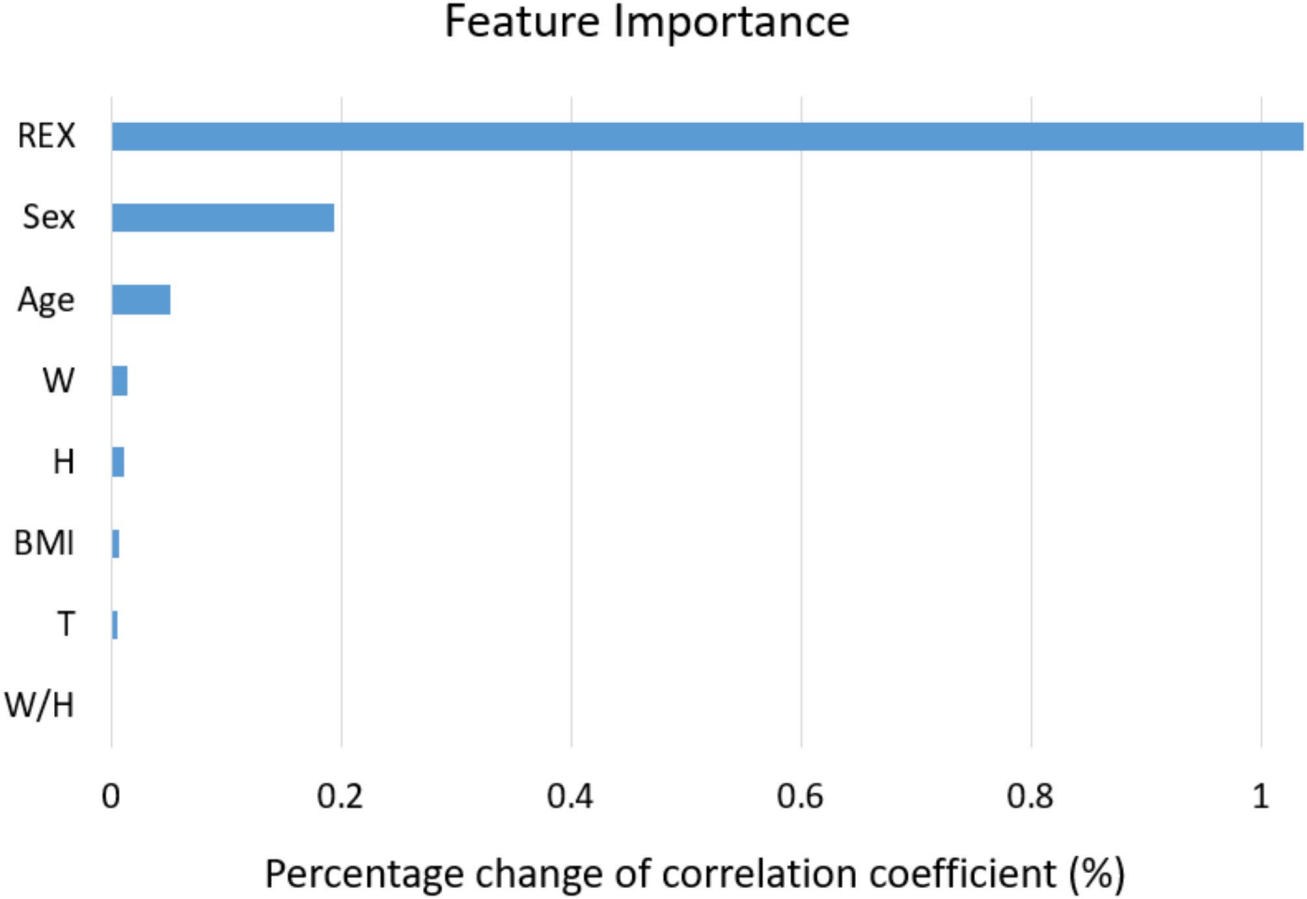
The rankings of feature importance determined by the permutation test for the ANN prediction model. REX = reached exposure; BMI = Body Mass Index; W = body weight; H = body height; T = chest thickness.

In the ANN model, the results of feature importance demonstrated that the REX and sex were top two important features, whereas the W/H had the least importance in predicting mAs, as shown in Fig. 2.

Table 6 shows the demographic characteristics and exposure parameters of the overexposure (REX > 355.6) and underexposure (REX < 355.6) groups. The comparisons showed that the overexposure group had significantly higher age, height, weight, W/H, BMI, body thickness, and mAs values than those of underexposure group.

**Table 6:** The demographic characteristics, exposure parameter and indicator of the overexposure (REX > 355.6) and underexposure (REX < 355.6) groups.

Number of patients	Age (years)	Height (cm)	Weight (kg)	W/H (kg/cm)	BMI (kg/m^2^)	Body thickness (cm)	mAs	Reached exposure (REX)
Overexposure (M/F = 478/11)	26.9 ± 7.7*	173.5 ± 6.1*	79.7 ± 11.8*	0.46 ± 0.06*	26.5 ± 3.6*	19.9 ± 3.6*	3.48 ± 1.20*	411.1 ± 41.6*
Underexposure (M/F = 437/74)	24.7 ± 6.2*	170.0 ± 6.7*	63.4 ± 9.5*	0.37 ± 0.05*	21.9 ± 3.0*	18.0 ± 3.6*	2.03 ± 0.65*	315.4 ± 26.6*

The data are expressed as mean ± standard deviation. Asterisks (*) indicate significant difference between the two groups.

**Fig. 3:**
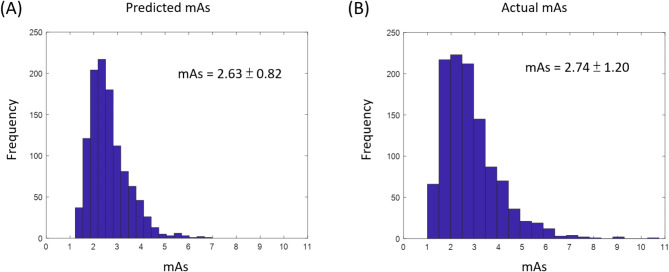
The histograms of the predicted (**A**) and actual mAs (**B**) in all subjects. The predicted mAs was derived using the ANN model with target exposure indicator (REX = 355.6), whereas the actual mAs was determined by AEC.

By using the ANN model with target exposure indicator (REX = 355.6), the results showed that the predicted mAs values were significantly lower than the values determined by AEC (2.63 ± 0.82 vs. 2.74 ± 1.20 mAs) for all patients. Figure 3A and B show the histograms of the predicted mAs values obtained using the ANN model with target exposure indicator (REX = 355.6) and the actual mAs values determined by AEC for all patients.

**Fig. 4:**
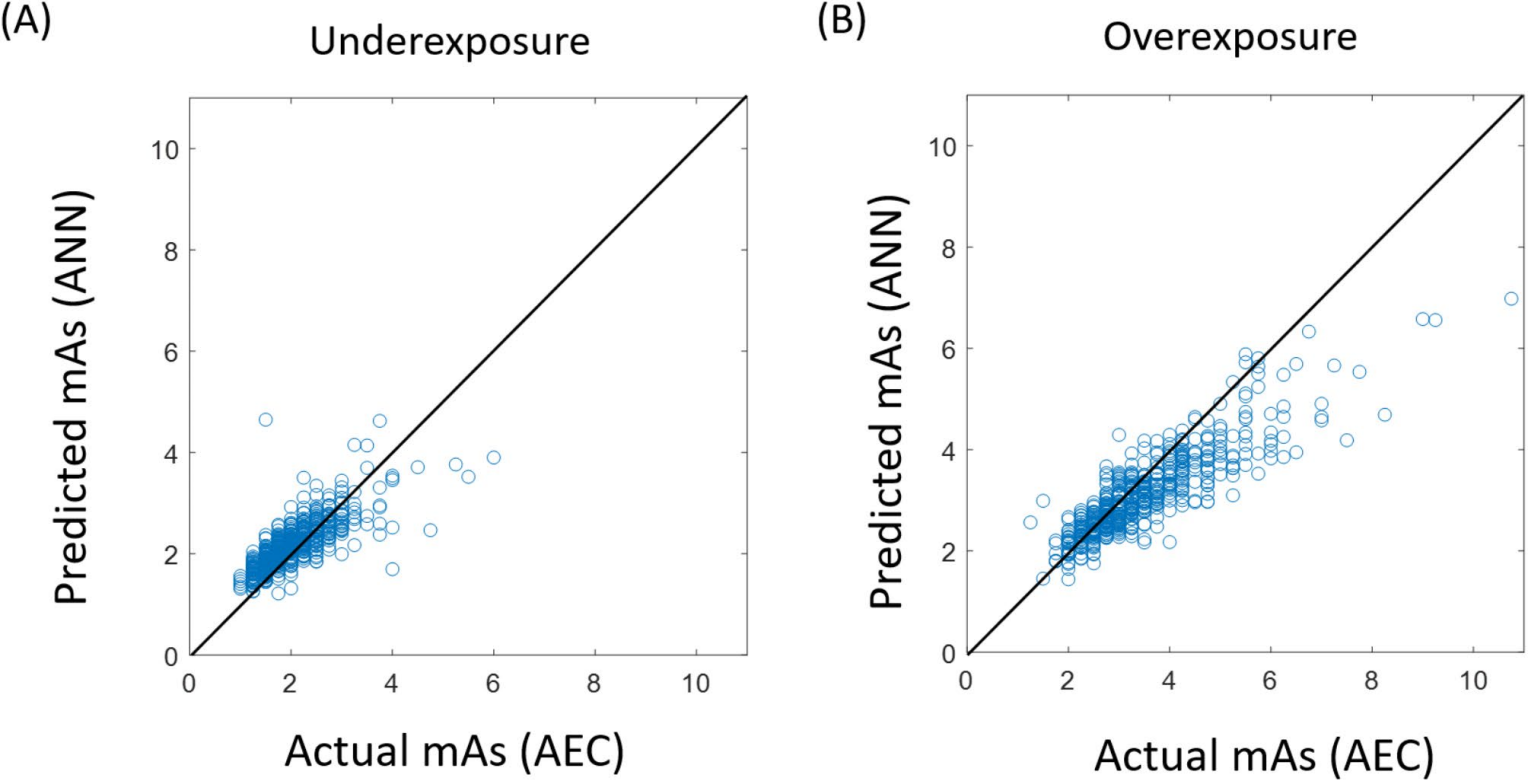
The relationship between the predicted mAs values derived using the ANN model with target exposure indicator and the actual mAs determined by AEC in the (**A**) underexposure (REX < 355.6) and (**B**) overexposure (REX > 355.6) groups.

In the overexposure group (REX > 355.6), the results demonstrated that the predicted mAs values were significantly lower than the values determined by AEC (3.12 ± 0.83 vs. 3.47 ± 1.20 mAs, about 10%) in the overexposure group. However, the predicted mAs values were significantly higher than the values determined by AEC (2.19 ± 0.49 vs. 2.03 ± 0.65 mAs, about 8%) in the underexposure group (REX < 355.6). The relationship between the predicted and actual mAs values in the underexposure and overexposure are shown in Fig. 4A and B, respectively. The results demonstrated that the ANN model with target exposure indicator helped increase and decrease mAs values for the underexposure and overexposure groups, respectively.

**Fig. 5:**
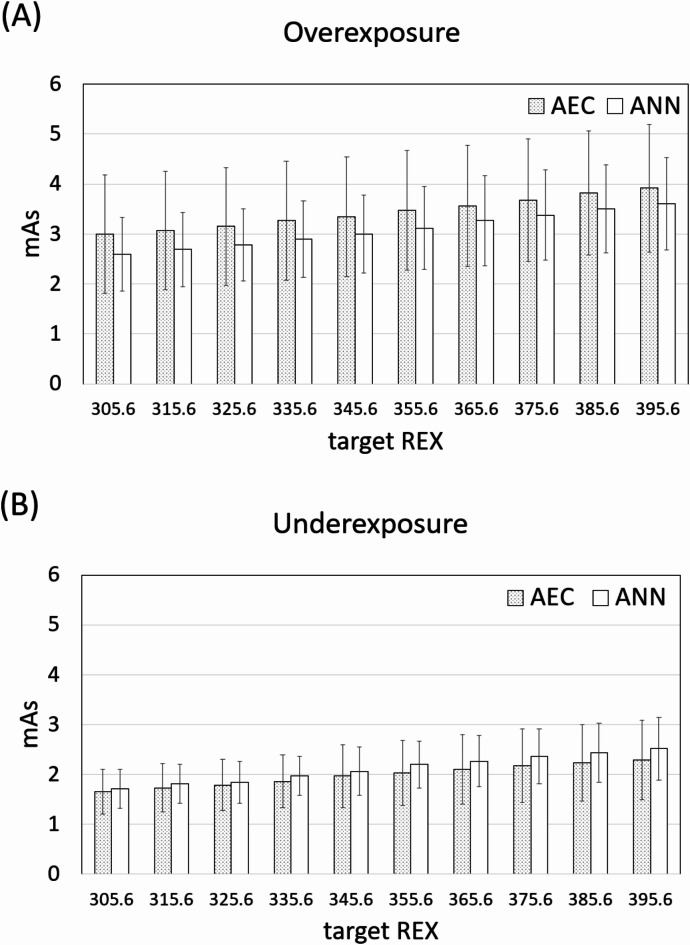
The differences between the predicted mAs values derived using the ANN model and the actual mAs determined by AEC in the (**A**) overexposure and (**B**) underexposure groups at different target REX values.

Moreover, the comparison further demonstrated that when lowering the target REX from 395.6 to 305.6, the radiation dose was reduced from 7.9 to 13.2% in the overexposure group as compared with those determined by AEC. However, the radiation dose was increased from 3.4 to 10.1% in the underexposure group when increasing the target REX from 305.6 to 395.6, as shown in Fig. 5. Overall, the prediction model increased radiation dose by 1.9% and 3.8% in all subjects when REX values were 385.6 and 395.6, respectively; however, the radiation dose was reduced from 0.2 to 11.7% in all subjects when lowering target REX from 375.6 to 305.6.

## Discussion

Establishing the target exposure value is crucial for optimizing image quality while minimizing the radiation dose to patients undergoing radiographic examinations^22^. Because radiographic image quality was associated with different factors, including anatomy-specific examinations, exposure factors (i.e., kVp and mAs), body parameters (i.e. W, H, W/H, BMI, and T), detector types, and vendors/systems, some studies have investigated the relationship between body parameters, exposure parameters, and image quality, which are correlated with each other^1–7^. To the best of our knowledge, no previous study has used ML to construct AI models for predicting mAs in chest radiography. Therefore, this study used the ML approach to establish an AI model for predicting an appropriate mAs with target exposure indicator and non-contact thickness measurement. In our study, the target exposure indicator, which serves as a measure of physical image quality, was used as an input to predict the appropriate mAs required to produce a radiograph with the desired image quality. Additionally, non-contact thickness measurement was performed to accurately determine the average chest thickness for constructing the prediction model. Our findings demonstrated that the ML models accurately predicted mAs values for patients undergoing chest digital radiography prior to the procedure.

To establish the target exposure indicator for chest radiography, an anthropomorphic chest phantom was exposed five times to obtain an average REX value. In line with the results of a previous study, this study showed that the average REX value was 355.6 ± 8.0^24^. Following the phantom study, ML was employed to establish AI models for predicting mAs values using AEC. The comparisons revealed that the ElmanNet ANN model performed the best in predicting the mAs values. The findings indicated that the ML approach was feasible for constructing AI models to accurately predict mAs values in patients undergoing chest radiography. Moreover, the three best ML models for predicting mAs values were the ANN, BAG, and RF models. The performance of the ANN model was superior to other models, with higher correlation coefficients (0.906 ± 0.003), lower RMSE (0.187 ± 0.002), and lower MAE (0.352 ± 0.005) values in the testing dataset. Although ANN model required relatively longer training time (1.72 ± 0.07 s) than other models, the ANN model was considered the final model for predicting the mAs values for chest radiography. Furthermore, the feature importance test conducted on the ANN model identified REX values and sex as the two most significant features. This suggests that physical image quality and body composition have a substantial impact on the accuracy of predicted mAs in chest radiography. It is known that male subjects have more lean muscle and less body fat than female subjects^34^. Besides, in the chest region, the breast tissue of female subjects could additionally absorb X-ray photons as compared to a same-sized male subject, so less X-ray photons were able to penetrate thorax and reach the AEC sensor. As a result, more X-ray photons (or higher mAs) were generated before the tube current was terminated by AEC technique. In contrast, W/H and thickness were found to be the least two influential features in predicting mAs. It is known that body thickness is an important factor to adjust radiological exposure in most cases during radiography. However, the thoracic cavity consists of air-filled lungs, soft tissues, and bones, so the chest thickness alone may not properly reflect the composition of the chest. Further investigation will be needed to understand the role of thickness in predicting mAs for other body part radiography.

In the overexposure group (REX > 355.6), it was noted that the height, weight, BMI, and body thickness were significantly higher than those in the underexposure group (REX < 355.6), indicating that the patients with larger body size and thickness may be overexposed during chest radiography with AEC. After applying the ANN model with target exposure indicator (REX = 355.6), the predicted mAs values were on average 10% less than the values determined by AEC (3.12 vs. 3.47 mAs) in the overexposure group. Conversely, in the underexposure group, the predicted mAs values were about 8% higher than the values determined by AEC (2.19 vs. 2.03 mAs). In addition to the average target exposure indicator (REX = 355.6), the present study further compared the difference of radiation exposure between the predicted mAs and those determined by AEC at different target REX values ranging from 305.6 to 395.6. The results demonstrated that the radiation exposure was reduced by the prediction model as the target REX values were lowered. At target REX = 305.6, although the radiation exposure was increased by 3.4% in the underexposure group, it was reduced by about 13.2% and 11.7% in the overexposure group and all patients, respectively. These findings suggest that the mAs prediction model helps predict appropriate mAs for chest radiography to achieve user-defined target exposure indicator. Moreover, the proposed method could be used as an adjunct to AEC technique to determine an appropriate upper limit of mAs, so that the radiation exposure remained unchanged for those under-exposed patients. In patients where AEC may not function optimally, such as those with metal implants, the proposed method might be a potential alternative for determining an appropriate patient-specific mAs. Further investigation is required to assess the extent to which our proposed method can reduce unnecessary radiation for patients with metal implants, in comparison to the use of AEC. Because chest radiography used low exposure technique, dose reduction of 0.3 mAs (or 10%) was relatively small. For other body parts, such as kidney-ureter-bladder, the radiography requires higher exposure technique (up to 30–120 mAs), so the 10% exposure reduction could be more significant in terms of cancer risk.

In a previous study, a method was established to determine the optimal exposure by using the target exposure index and approximating patient thickness based on the W/H ratio. The prediction method employed a first-order exponential function that considered body thickness, entrance-surface air kerma, and the grid Bucky factor. The results of the study demonstrated a significant reduction in both the entrance-surface dose and the dose-area product for patients undergoing radiography^1^. Although this method could be applied to chest, abdomen, and pelvis radiography, the study did not show the relationship between the actual and predicted mAs for each type of examination. Other previous studies explored the relationship between body parameters and exposure parameters using correlation analysis and curve fitting approaches^2,6,7^, and demonstrated that different body parameters exhibited varying degrees of correlation with exposure parameters in radiography. In the present study, a ML approach was employed to build an AI model for predicting the appropriate mAs by integrating various body parameters and the target exposure indicator. Our study yielded a correlation coefficient of 0.906 between the predicted and actual mAs in the ANN model, indicating a high level of linearity between the predicted and the actual mAs. Additionally, we utilized a novel non-contact infrared sensor to measure two-dimensional chest thickness, which was demonstrated to have excellent accuracy in distance measurement. This precise and reliable measurement of chest thickness may further enhance the accuracy of the model in predicting mAs. Furthermore, the target exposure indicator derived from chest phantom radiography proved to be effective in minimizing both overexposure and underexposure in chest radiography. This finding highlights the potential for the target exposure indicator to serve as a valuable tool for optimizing radiation exposure in clinical practice.

This study has some limitations that need to be considered. First, because the prediction models were established using chest radiography datasets acquired using a digital radiography system, they may not be used for other anatomy-specific examinations and on different radiography systems. Thus, constructing AI models for predicting mAs for other anatomical examinations by collecting a new dataset or using transfer learning is required. Second, the vendor-specific exposure indicator provided by the manufacturer is REX value, so the prediction model needs to be retrained using different exposure indicators, such as EI, lgM, DI, and S values. Third, the enrolled subjects were mainly male; therefore, the prediction model may have been biased because of the imbalance between male and female subjects. Further investigation will be needed to build a robust prediction model using a fully balanced dataset. Fourth, the study cohort was relatively young and only consisted of a small number of obese patients (BMI ≥ 30 kg/m^2^) (only 8.4%); therefore, the prediction model may not be accurate for elderly or obese patients. Finally, the prediction model was constructed for adult patients aged > 20 years; therefore, the model must be retrained for pediatric patients by using the target exposure indicator derived from a pediatric chest phantom.

## Conclusions

This study used the ML approach to construct AI models for predicting mAs values based on patient body parameters. The results demonstrated that in the best prediction model, the correlation coefficients between the predicted and actual mAs values were 0.906. By controlling for the target exposure indicator in the prediction model, this study showed that the ANN model could predict appropriate mAs and was effective in minimizing both overexposure and underexposure in chest radiography. Therefore, we concluded that the ML approach was feasible for constructing an AI model for predicting appropriate mAs with target exposure indicator for chest digital radiography.

## Electronic supplementary material

Below is the link to the electronic supplementary material.

Supplementary Material 1

## Data Availability

The datasets generated and/or analysed during the current study are not publicly available due the privacy regulation of local institutional review board but are available from the corresponding author on reasonable request.

## Code availability

The MATLAB code of model training and testing for this study is included as a supplementary document.
